# Neutral and charged boron-doped fullerenes for CO_2_ adsorption

**DOI:** 10.3762/bjnano.5.49

**Published:** 2014-04-07

**Authors:** Suchitra W de Silva, Aijun Du, Wijitha Senadeera, Yuantong Gu

**Affiliations:** 1 School of Chemistry, Physics and Mechanical Engineering, Queensland University of Technology, Brisbane 4001, Australia

**Keywords:** adsorption, boron doping, CO_2_ capture, density functional theory (DFT), heterofullerene

## Abstract

Recently, the capture and storage of CO_2_ have attracted research interest as a strategy to reduce the global emissions of greenhouse gases. It is crucial to find suitable materials to achieve an efficient CO_2_ capture. Here we report our study of CO_2_ adsorption on boron-doped C_60_ fullerene in the neutral state and in the 1*e*^−^-charged state. We use first principle density functional calculations to simulate the CO_2_ adsorption. The results show that CO_2_ can form weak interactions with the BC_59_ cage in its neutral state and the interactions can be enhanced significantly by introducing an extra electron to the system.

## Introduction

The continuous dependence on fossil fuel combustion for the generation of energy has dramatically increased the atmospheric CO_2_ concentrations over the last century. Despite concerns for global climatic changes and many attempts to sustainably generate energy, fossil fuel combustion continues to be the main source of electricity while releasing 13 Gt of CO_2_ [1] to the atmosphere each year. Therefore CO_2_ capture and storage (CCS) technology is a promising solution to reduce atmospheric CO_2_ emissions [2]. Solvent absorption that is based on amines is the most common technology for the capture of CO_2_. However this method is criticized for its very high energy consumption and operational limitations such as corrosion, slow uptake rates, foaming and large equipment. Hence there is a huge interest in solid adsorbent materials for CCS [3–6]. In past few years metal organic frameworks (MOFs) have emerged as solid CO_2_ adsorbent materials due to their tuneable chemical and physical properties.

Particularly, there is growing interest for metal free carbon-based nanomaterials for gas adsorption. Carbon-based nanomaterials such as fullerene, carbon nanotubes and graphene offer excellent thermal and chemical stability as CO_2_ adsorbents [7–8]. Heterofullerenes are fullerene structures in which one or more cage carbon atoms are substituted by heteroatoms [9]. In addition to the properties mentioned above, which are inherent to carbon-based nanomaterials, heterofullerenes also offer excellent tuneable chemical and physical properties [10]. Gas adsorption on heterofullerenes is an appealing subject. B. Gao et al. [11] studied CO_2_ adsorption on calcium decorated C_60_ fullerene and F. Gao et al. [12] studied O_2_ adsorption on nitrogen-doped fullerene.

Boron-doped C_60_ fullerenes are one of the most structurally stable heterofullerenes [9]. Guo et al. synthesized B-doped C_60_ fullerenes for the first time, in microscopic amounts by laser vaporisation [13]. Zou et al. [14] demonstrated the synthesis of B-doped C_60_ fullerene by using radio frequency plasma-assisted vapour deposition. Recently Dunk et al. [15] introduced a method to produce BC_59_ directly from exposing C_60_ fullerene to boron vapour. Wang et al. [16] stated that substituting a single C atom of the C_60_ fullerene with a B atom does not cause a significant distortion in the cage structure. The net change in the dihedral angle due to the doping is only 1.6% and Kurita et al. [17] predicted that due to the similarity between the C–B bond and the C–C bond, the changes in the bond lengths are less than 5%. Therefore the BC_59_ fullerene has a similar structural and thermal stability as C_60_ fullerene. Despite the numerous study results, which confirm the structural stability of B-doped C_60_ fullerene, very little studies have been done on applications of B-doped fullerene. Here, for the first time we report a study about the CO_2_ adsorption on B-doped C_60_ fullerene, in which a single C atom is replaced with a B atom.

Sun et al. [8] predicted an enhanced CO_2_ adsorption on 1*e*^−^- and 2*e*^−^-charged boron nitride sheets and nanotubes, which show very little chemical affinity towards CO_2_ in their neutral state. Also Sun et al. [18] showed that chemical interactions between boron–carbon nanotubes (B_2_CNT) and CO_2_ can be enhanced by introducing extra electrons to the system. The enhanced interaction of CO_2_ with adsorbent materials by electron injection has been further proved by Jiao et al. [19]. Therefore, we will investigate the CO_2_ adsorption on BC_59_ fullerene in both the neutral and the 1*e*^−^-charged states.

## Computational Details

First-principles density functional theory (DFT) calculations were carried out to study CO_2_ adsorption on the BC_59_ cage. The BC_59_ structure was fully optimized in the given symmetry. The calculations were carried out at B3LYP [20–22] level of theory while using the split valance polarized basis set 6-31G(d). B97d [23–24] with the same basis set was used for calculations when non-covalent interactions are predominant. The CO_2_ adsorption on BC_59_ was studied in the neutral state and in the 1*e*^−^-charged state. The electron distribution and transfer were analysed with Mulliken population analysis method [25]. The adsorption energies were calculated using the following equation.

[1]
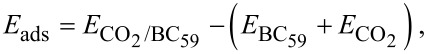


where *E*_ads_ is the adsorption energy, 
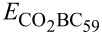
 is the total energy of the BC_59_ cage with a CO_2_ molecule adsorbed and 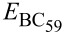 and 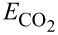 are the energies of the isolated BC_59_ cage and CO_2_ molecule, respectively. For a favourable adsorption the calculated adsorption energy should have a negative value. To provide more accurate results for the chemisorption energy the counterpoise corrected energy [26–27] was also calculated.

The transition state was located by using the synchronous transit-guided quasi-Newton (STQN) method [28–29], which was then fully optimized by using the Berny algorithm at the B3LYP/6-31G(d) level. The optimized transition structure was used for IRC calculations at the same level of theory [30–31]. All calculations were carried out by using the Gaussian 09 package [32]. The GaussView 5 package [33] was used to visualize the optimized molecular structures, molecular orbitals and charge distributions.

## Results and Discussion

**Figure 1:**
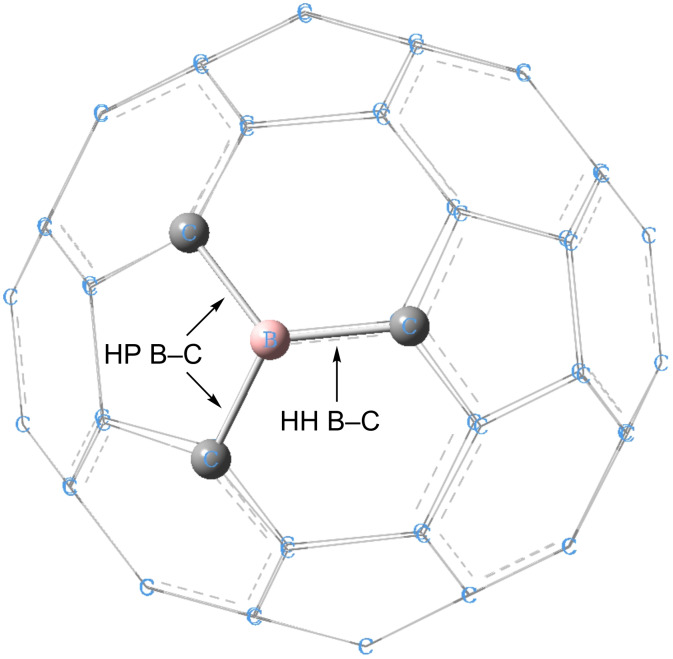
Sites for CO_2_ adsorption on BC_59_. The B and C atoms of HH B–C and HP B–C sites are represented as ‘ball and bond’-type and the rest of the atoms are represented as ‘wireframes’. Atom colour code: grey, carbon; pink, boron.

The substitution of a C atom in the C_60_ fullerene by a B atom causes a charge transfer between C and B atoms, which results in an unbalanced charge distribution in the fullerene cage. The unbalanced charge distribution forms B–C complex sites for the adsorption of CO_2_ (Figure 1). Here we considered two possible sites for the CO_2_ adsorption: the B–C atomic site between two hexagonal rings (HH B–C site) and two identical B–C sites between a hexagonal ring and pentagonal ring (HP B–C site).

### Adsorption of CO_2_ on uncharged BC_59_ fullerenes

**Figure 2:**
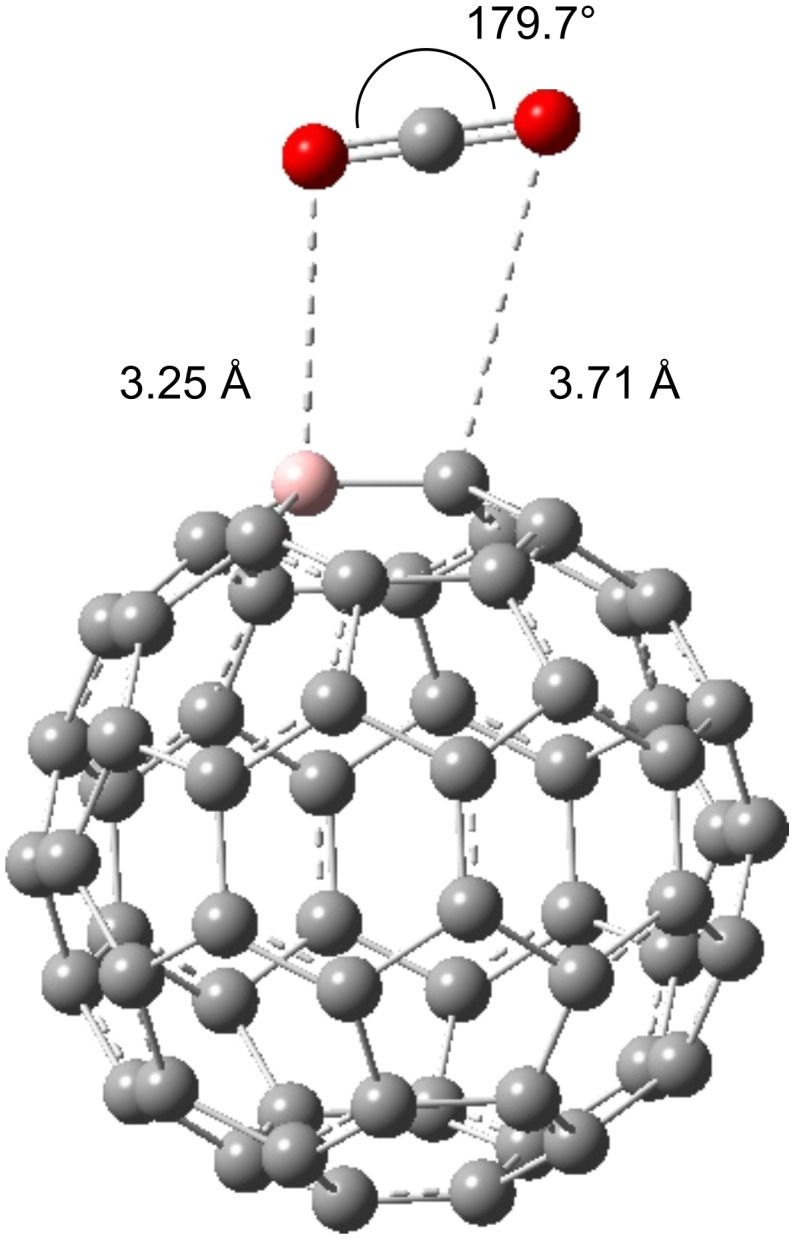
Configuration of physisorbed CO_2_ on neutral BC_59_. Atom colour code: grey, carbon; pink, boron; red, oxygen.

According to our simulation results, the CO_2_ molecules can only form weak interactions with BC_59_ cage in its neutral state. The physisorption energy is a weak −2.04 kcal/mol (−4.1 kcal/mol for B97D/6-31G(d) calculations) and the weak interactions are mainly van der Waals interactions between the CO_2_ molecule and the adsorbent. The CO_2_ physisorbed configuration is shown in Figure 2. The CO_2_ molecule sits parallel to the boron–carbon plane of the BC_59_ fullerene cage. The B^…^O and C^…^O bond distances are 3.25 Å and 3.71 Å, respectively. The CO_2_ molecule undergoes very slight structural changes upon physisorption on the uncharged BC_59_ fullerene cage. The O–C–O angle is slightly bent to 179.7° and the changes to the C=O bond lengths are negligibly small. The doped fullerene cage hardly undergoes any structural change. The charge transfer between CO_2_ and BC_59_ is only 0.008*e*.

### Effects of charges on the structure

**Figure 3:**
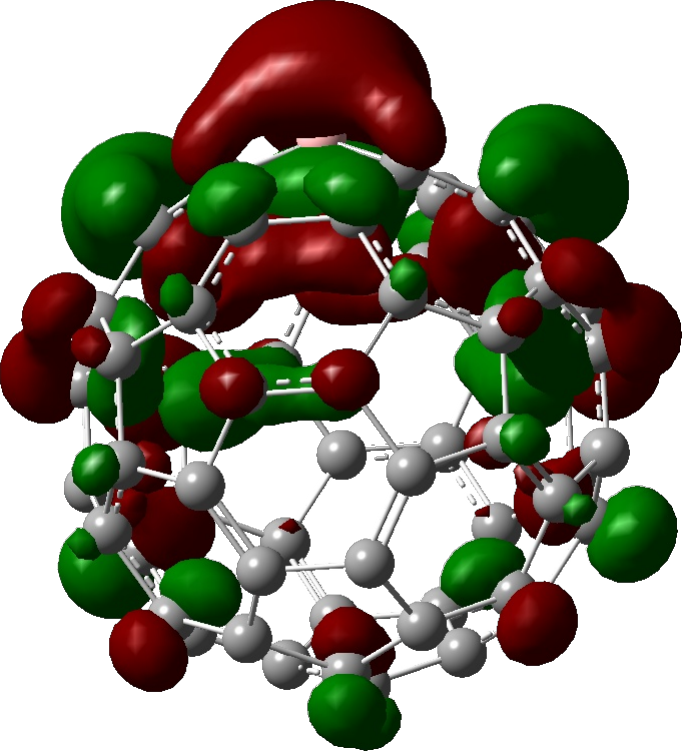
LUMO of neutral BC_59_. The orbitals are drawn at an isosurface value of 0.02. The colours of the orbitals: red, positive wave function; green, negative wave function. Atom colour code: pink, boron; grey, carbon.

**Figure 4:**
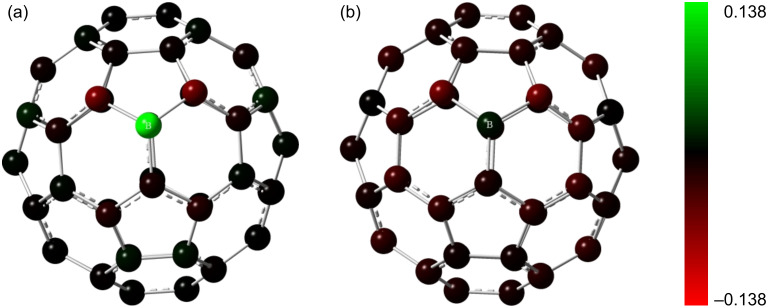
Mulliken charge distribution of (a) neutral BC_59_ and (b) 1*e*^−^-BC_59_. The atoms are shaded based on the charge distribution on each atom. The comparison suggests that the most notable charge transfer is on the B atom.

Kim et al. [34] predicted that C_59_B^−^ should be a stable entity because of the isoelectronic configuration with C_60_. This claim is further validated by experimental observations by Dunk et al. [15]. The Mulliken charge analysis and the electron density distributions of the lowest unoccupied molecular orbitals (LUMO) are adopted to assess the influence of changing the charge state of BC_59_. Figure 3 shows that the LUMO of the neutral BC_59_ is noticeably concentrated on the B atom and the neighbouring C atoms. Furthermore experimental results of Guo et al. [13] showed that boron doping creates an electron defficient site at the B atom. This suggests that an additional electron added to the system will be accepted by the B atom. This hypothesis is consistent with theoretical predictions of Kurita et al. [17] and Xie et al. [35], who stated that the doped B atom in C_60_ fullerene acts as an electron acceptor. The comparison of the Mulliken population analysis of the neutral and the 1*e*^−^-state of BC_59_ proves that the negative charge introduced to the system is essentially accepted by the B atom. The Mulliken atomic charge of the B atom in the BC_59_ structure in the neutral state has changed from 0.138 to 0.012 upon the introduction of the negative charge, while as shown in Figure 4 the charges on the C atoms are not changed significantly.

### CO_2_ adsorption on BC_59_ fullerene in the 1*e*^−^-state

Next we studied the CO_2_ adsorption on a 1*e*^−^-charged BC_59_ cage. The results confirm that the negatively charged BC_59_ fullerene exhibits a stronger interaction with CO_2_. Unlike the neutral BC_59_, for which the interaction with CO_2_ molecule was only physical, here the charged BC_59_ forms a substantial chemical interaction with CO_2_ causing the molecule to undergo significant structural deformations. A stable CO_2_ adsorption is observed at the HH B–C site. The chemisorption energy of −15.41 kcal/mol (−64.48 kJ/mol) (−13.48 kcal/mol with BSSE correction) agrees well with the ideal range of chemisorption energy (40–80 kJ/mol) for a good CO_2_ adsorbent [36].

**Figure 5:**
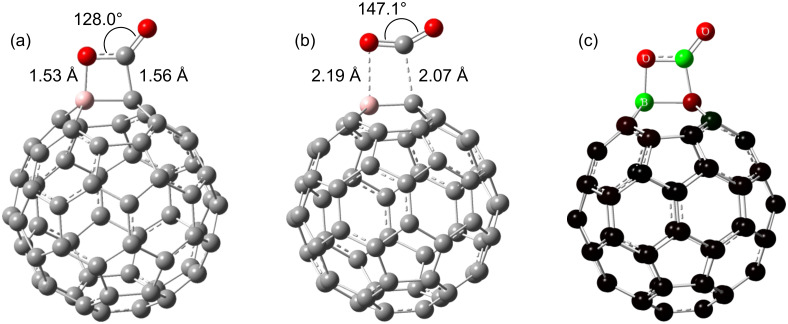
(a) CO_2_ chemisorption and (b) transition structure for CO_2_ chemisorption on 1*e*^−^-charged BC_59_. Atom colour code: grey, carbon; pink, boron; red, oxygen. (c) Charge distribution after CO_2_ chemisorption.

The CO_2_ molecule undergoes considerable distortion upon chemically adsorbing on the 1*e*^−^-charged BC_59_ fullerene. A C=O bond of the CO_2_ molecule is broken when one oxygen atom forms a bond with the boron atom (which will be referred as O_a_ in the following discussion and the other oxygen atom as O_b_) and the C atom of the CO_2_ molecule forms a bond with the C atom on the HH B–C site of the cage structure. The linear O–C–O bond of CO_2_ is bent to 128.0° in the adsorbed form. The C=O_b_ bond which is originally 1.169 Å (experimentally 1.162 Å [37]) is elongated to 1.208 Å, while the length of the C–O_a_ bond is expanded to 1.336 Å.The adsorption site of the BC_59_ fullerene also undergoes considerable stretching. The HH B–C site is protruded outwards by about 0.05 Å. The B–C bond of the HH B–C site has stretched from 1.496 to 1.672 Å. The Mulliken population analysis shows that a charge transfer of 0.42 has occurred from the BC_59_ fullerene to the CO_2_ molecule. Comparison of the charge distribution on BC_59_^−^ before (Figure 4b) and after (Figure 5c) CO_2_ adsorption, confirms that the injected electron is occupied by the CO_2_ molecule.

The higher adsorption energy and the significant distortions in the structure confirm a stronger interaction between CO_2_ molecule and negatively charged BC_59_ than its neutral state. These interactions can be explained due to the Lewis acidity of CO_2_, which prefers to accept electrons [18]. On the other hand the B atom of the BC_59_ becomes less positively charged upon the addition of an extra electron. Therefore it becomes more likely to donate electrons to the CO_2_ molecule leading to stronger interactions between the two molecules.

**Figure 6:**
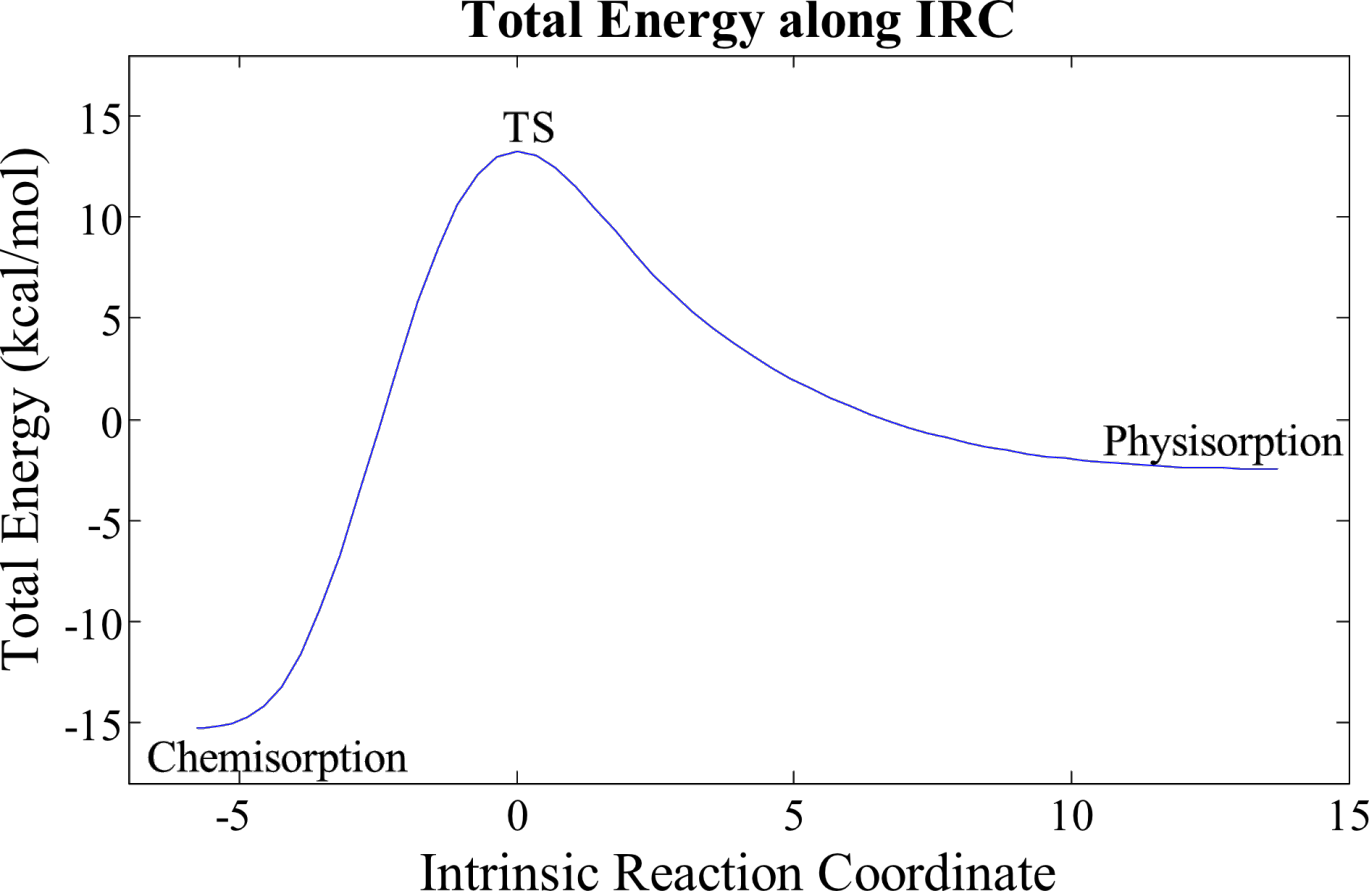
Intrinsic reaction pathway for CO_2_ chemisorption on 1*e*^−^-charged BC_59_ from the physisorbed configuration. The total energy = 0 point corresponds to the total energy of *E*_CO2_ + *E*_BC59_^−1^.

Figure 6 shows the minimum energy pathway for the adsorption from the physisorbed state to the chemisorbed configuration. We performed frequency calculations on the optimized transition structure, which confirms that it is a first order saddle point and hence an actual transition structure. From this figure, the activation barrier for the chemisorption is estimated to be 13.25 kcal/mol (55.43 kJ/mol). The low barrier of the reaction indicates that the reaction is energetically favourable.

For the desorption step, the removal of the added charge will decrease the stability of the bond between CO_2_ and the doped fullerene. The thermodynamic analysis of the reaction shows that the CO_2_ chemisorption is spontaneous only for temperatures less than 350 K. Therefore we suggest a method of manipulating the charge state and the temperature of the system for adsorbent recycling. Charging the system can be achieved by electrochemical methods, electrospray, and electron beam or gate voltage control methods [8].

## Conclusion

By using DFT calculations we have studied the adsorption mechanisms of CO_2_ on a C_60_ fullerene cage, in which a single C atom is substituted by a B atom. Our calculation results show that the BC_59_ cage, in its neutral state, shows a low chemical interaction with CO_2_ molecule, which only physisorbs with *E*_ads_ = −2.04 kcal/mol. However CO_2_ adsorption on the BC_59_ can be significantly enhanced by injecting negative charges into the structure. The CO_2_ molecule chemisorbs on the 1*e*^−^-charged BC_59_ with *E*_ads_ = −15.41 kcal/mol. This study suggests that we can conclude 1*e*^−^-charged BC_59_ cage structure is a promising CO_2_ adsorbent.
